# Mortality and critical care unit admission associated with the SARS-CoV-2 lineage B.1.1.7 in England: an observational cohort study

**DOI:** 10.1016/S1473-3099(21)00318-2

**Published:** 2021-11

**Authors:** Martina Patone, Karen Thomas, Rob Hatch, Pui San Tan, Carol Coupland, Weiqi Liao, Paul Mouncey, David Harrison, Kathryn Rowan, Peter Horby, Peter Watkinson, Julia Hippisley-Cox

**Affiliations:** a Nuffield Department of Primary Care Health Sciences, University of Oxford, Oxford, UK; b Nuffield Department of Clinical Neurosciences, University of Oxford, Oxford, UK; c Nuffield Department of Medicine, University of Oxford, Oxford, UK; d Intensive Care National Audit & Research Centre, London, UK; e Division of Primary Care, School of Medicine, University of Nottingham, Nottingham, UK; f Department of Medical Statistics, London School of Hygiene and Tropical Medicine, London, UK; g Department of Health Services Research and Policy, London School of Hygiene and Tropical Medicine, London, UK; h Oxford University Hospitals NHS Foundation Trust and NIHR Biomedical Research Centre, Oxford, UK

## Abstract

**Background:**

A more transmissible variant of SARS-CoV-2, the variant of concern 202012/01 or lineage B.1.1.7, has emerged in the UK. We aimed to estimate the risk of critical care admission, mortality in patients who are critically ill, and overall mortality associated with lineage B.1.1.7 compared with non-B.1.1.7. We also compared clinical outcomes between these two groups.

**Methods:**

For this observational cohort study, we linked large primary care (QResearch), national critical care (Intensive Care National Audit & Research Centre Case Mix Programme), and national COVID-19 testing (Public Health England) databases. We used SARS-CoV-2 positive samples with S-gene molecular diagnostic assay failure (SGTF) as a proxy for the presence of lineage B.1.1.7. We extracted two cohorts from the data: the primary care cohort, comprising patients in primary care with a positive community COVID-19 test reported between Nov 1, 2020, and Jan 26, 2021, and known SGTF status; and the critical care cohort, comprising patients admitted for critical care with a positive community COVID-19 test reported between Nov 1, 2020, and Jan 27, 2021, and known SGTF status. We explored the associations between SARS-CoV-2 infection with and without lineage B.1.1.7 and admission to a critical care unit (CCU), 28-day mortality, and 28-day mortality following CCU admission. We used Royston-Parmar models adjusted for age, sex, geographical region, other sociodemographic factors (deprivation index, ethnicity, household housing category, and smoking status for the primary care cohort; and ethnicity, body-mass index, deprivation index, and dependency before admission to acute hospital for the CCU cohort), and comorbidities (asthma, chronic obstructive pulmonary disease, type 1 and 2 diabetes, and hypertension for the primary care cohort; and cardiovascular disease, respiratory disease, metastatic disease, and immunocompromised conditions for the CCU cohort). We reported information on types and duration of organ support for the B.1.1.7 and non-B.1.1.7 groups.

**Findings:**

The primary care cohort included 198 420 patients with SARS-CoV-2 infection. Of these, 117 926 (59·4%) had lineage B.1.1.7, 836 (0·4%) were admitted to CCU, and 899 (0·4%) died within 28 days. The critical care cohort included 4272 patients admitted to CCU. Of these, 2685 (62·8%) had lineage B.1.1.7 and 662 (15·5%) died at the end of critical care. In the primary care cohort, we estimated adjusted hazard ratios (HRs) of 2·15 (95% CI 1·75–2·65) for CCU admission and 1·65 (1·36–2·01) for 28-day mortality for patients with lineage B.1.1.7 compared with the non-B.1.1.7 group. The adjusted HR for mortality in critical care, estimated with the critical care cohort, was 0·91 (0·76–1·09) for patients with lineage B.1.1.7 compared with those with non-B.1.1.7 infection.

**Interpretation:**

Patients with lineage B.1.1.7 were at increased risk of CCU admission and 28-day mortality compared with patients with non-B.1.1.7 SARS-CoV-2. For patients receiving critical care, mortality appeared to be independent of virus strain. Our findings emphasise the importance of measures to control exposure to and infection with COVID-19.

**Funding:**

Wellcome Trust, National Institute for Health Research Oxford Biomedical Research Centre, and the Medical Sciences Division of the University of Oxford.

## Introduction

On Sept 20, 2020, a new variant of SARS-CoV-2—known as variant of concern 202012/01, or lineage B.1.1.7—was detected by the COVID-19 Genomics UK consortium in England.^1^ Lineage B.1.1.7 has multiple changes, including an N501Y (Asn501Tyr) substitution in the spike protein that enhances binding to the human ACE2 receptor, which the virus uses to enter the cell.^2^ These changes have been suggested to result in increased infectivity, with initial reports of 50–74% increased transmissibility.^3^ Early analyses of mortality linked to diagnostic data have suggested that infection with lineage B.1.1.7 might be associated with a higher risk of mortality compared with infection with other virus variants.^4^, ^5^, ^6^, ^7^, ^8^, ^9^ By contrast, a more recent study found no association between mortality and B.1.1.7 for patients admitted to hospital.^10^ This new finding, which appears to be discordant from the previous ones, has supported the idea that B.1.1.7 is not linked to severe disease or death. However, this result rather suggests that the effect of B.1.1.7 is different in a hospitalised cohort than in the general population and does not exclude an increased risk of hospital admission with the new lineage. Effects of B.1.1.7 on critical care admission or outcomes are still unknown.

**Research in context**

**Evidence before this study**

A new variant of SARS-CoV-2, variant of concern 202012/01 or lineage B.1.1.7, was detected in England in September 2020. Lineage B.1.1.7 has been associated with increased transmissibility. Early analyses have suggested infection with lineage B.1.1.7 might be associated with a higher risk of mortality compared with infection with other virus lineages, but these analyses had either limited ability to adjust for key confounding variables or did not consider admission to a critical care unit (CCU). The effects of lineage B.1.1.7 on severe COVID-19 outcomes remain unclear. We searched PubMed for articles published between Sept 1, 2020, and April 15, 2021, containing “B.1.1.7”, “B117”, “VOC-202012/1”, “UK variant”, “Kent variant”, “sgtf” or “S gene target”, in combination with at least one of “mortality”, “ICU”, “fatality”, “severity”, “critical care” or “hospital” in any language. We found 36 articles. Many of these focused on the sensitivity of lineage B.1.1.7 to vaccine antibodies or on the increased transmissibility and mortality of the lineage. We found no peer-reviewed publications on the effects of lineage B.1.1.7 on critical care admission and mortality and only one study that estimated mortality while controlling for patients' demographic and clinical outcomes.

**Added value of this study**

Our study found a 65% higher risk of 28-day mortality associated with infection with lineage B.1.1.7 in patients tested in the community compared with patients infected with non-B.1.1.7 SARS-CoV-2, when adjusting for key confounding variables. The risk of CCU admission for patients with lineage B.1.1.7 was double the risk associated with non-B.1.1.7 SARS-CoV-2 infection. For patients receiving critical care, the infecting lineage was not associated with the risk of mortality at the end of critical care.

**Implications of all the available evidence**

Although we observed a higher risk of severe outcomes (CCU admission and overall mortality) associated with lineage B.1.1.7, once a patient was in a condition severe enough to be admitted to CCU, their risk of dying was not different between lineages. The higher overall mortality and rate of CCU admission associated with lineage B.1.1.7, combined with its known increased transmissibility, are likely to put health-care systems under additional stress.

In this study, we aimed to explore the association between lineage B.1.1.7 and the risk of receiving critical care and 28-day mortality, following a positive community SARS-CoV-2 test. Additionally, for patients with confirmed COVID-19 receiving critical care, we aimed to explore the association between lineage B.1.1.7 and receipt and duration of organ support in critical care, duration of critical care stay, and mortality at the end of critical care.

Previous analyses 4, 5, 6, 7, 8 had limited adjustment for key patient characteristics thought to be associated with COVID-19 outcomes. Therefore, the effect of lineage B.1.1.7 on severe COVID-19 outcomes, receipt of critical care, and mortality, when carefully adjusted for key patient characteristics, remains unclear. Such adjustments were possible in this study, given the availability of patient-level data on clinical and demographic characteristics in the datasets used.

## Methods

### Data platforms

For this observational cohort study, we used as main datasets the QResearch data platform and Intensive Care National Audit & Research Centre (ICNARC) COVID-19 study data. This analysis is part of a larger study protocol.^11^

QResearch is a high-quality research database based on records from 1350 primary care practices in England. Established in 2002, QResearch has been used extensively for epidemiological research.^12^ QResearch is one of the largest and most representative primary care research databases nationally,^13^ covering approximately 20% of the population of England. It has been used for COVID-19 research to inform the national pandemic response in the first pandemic wave.^14^, ^15^

The ICNARC COVID-19 study data, consisting of patients who are critically ill with confirmed COVID-19 (confirmed at admission to critical care), is hosted on the ICNARC Case Mix Programme (the national, high-quality clinical database for adult critical care) with complete coverage of critical care units across England, Wales, and Northern Ireland. For this study, only patients from England have been included.

The ethics approval for the development and validation of QResearch was granted by the East Midlands-Derby Research Ethics Committee (reference 18/EM/0400).

### Data linkage

To ensure that the QResearch and ICNARC COVID-19 study data platforms could be used to inform policy and planning during the UK COVID-19 epidemic, the primary care data and the critical care dataset were linked to other databases. The key data linkages for this research were COVID-19 testing data (the national registry of COVID-19 RT-PCR positive test results from Public Health England [PHE])—COVID-19 is a notifiable disease, and laboratories in England are required to send results of all tests to PHE—and Office of National Statistics (ONS) COVID-19 mortality data, which includes all deaths due to COVID-19 in England. The primary care dataset was additionally linked with the ICNARC COVID-19 study data, to derive the outcome variable for critical care unit (CCU) admission.

### Identification of B.1.1.7 by proxy

The molecular diagnosis of SARS-CoV-2 is included as part of test results sent to PHE. Lineage B.1.1.7 has a deletion of six nucleotides in the S gene that results in the deletion of two amino acids at positions 69 and 70 of the spike glycoprotein, which leads to S-gene molecular diagnostic assay failure (SGTF).

Cycle threshold (Ct) values for the S, N, and ORF1ab components of SARS-CoV-2, used to define the SGTF status, are available for COVID-19 RT-PCR positive tests taken in the community (but not hospital) setting. We defined SGTF as any test with non-detectable S gene and a Ct of 30 or lower for the N and ORF1ab targets, and we defined non-SGTF as any test with detectable S gene and Ct of 30 or lower for the N and ORF1ab targets. This definition was used by PHE in their report investigating the novel SARS-CoV-2 lineage B.1.1.7.^5^ All other tests were defined as inconclusive and excluded from the analysis. During the study period, in the UK, more than 99% of SGTFs were due to lineage B.1.1.7.^5^ Therefore, PCR-positive samples with SGTF were used as a proxy to identify the presence (or absence) of lineage B.1.1.7, and we used the SGTF status as defined to classify patients with the B.1.1.7 lineage and without in each cohort.

### Study cohorts and outcomes

We selected a start date of Nov 1, 2020, for the study on the basis of the emergence of lineage B.1.1.7, because 99% of patients with lineage B.1.1.7 were identified after this date (appendix p 2). We extracted two cohorts of patients from the linked data to explore the association of lineage B.1.1.7 with severe COVID-19 outcomes: the primary care cohort and the critical care cohort.

The primary care cohort comprised patients in primary care with a positive community COVID-19 test reported between Nov 1, 2020, and Jan 26, 2021, and known SGTF status. For this cohort, we extracted data for the following key patient characteristics: age (in years), sex (male or female), ethnic group (White, Indian, Pakistani, Bangladeshi, other Asian, Caribbean, Black African, Chinese, or other ethnic groups), body-mass index (BMI; <25, 25 to <30, 30 to <40, or ≥40), comorbidities (asthma, chronic obstructive pulmonary disease, type 1 and type 2 diabetes, and hypertension), smoking status (non-smoker, ex-smoker, and light, moderate, or heavy smoker), deprivation (quintiles, based on the Townsend score), housing category (care home, homeless, or neither), household size (one, two, three to five, or six or more people), and geographical region (ten regions across England).

The two outcomes of interest for the primary care cohort were CCU admission and 28-day mortality, with COVID-19 death defined as confirmed or suspected COVID-19 recorded on the death certificate, or death from any cause within 28 days of a positive COVID-19 test. For these outcomes, we obtained data from two different sources: the ICNARC COVID-19 study data platform for CCU admission and ONS mortality data for 28-day mortality. These two sources had different dates for last data updates. Therefore, patients were observed until Feb 7, 2021, for the CCU admission outcome (last data update of the ICNARC COVID-19 study data platform) and until Feb 23, 2021 for the 28-day mortality outcome (last ONS data update).

The critical care cohort comprised patients admitted for critical care with a positive community COVID-19 test reported between Nov 1, 2020, and Jan 27, 2021, and known SGTF status. The observational period for this cohort was between Nov 1, 2020, and Feb 7, 2021. For this cohort, we extracted data for the following key patient characteristics: age (in years), sex (male or female), ethnic group (White, Indian, Pakistani, Bangladeshi, other Asian, Caribbean, Black African, Chinese, or other ethnic groups), body-mass index (BMI; <25, 25 to <30, 30 to <40, or ≥40), deprivation (quintiles, based on the Townsend score), comorbidities (cardiovascular disease, respiratory disease, metastatic disease, and immunocompromised conditions), dependency (assistance with activities of daily living: none, some, or all), pregnancy (currently pregnant, recently pregnant in the past 6 weeks, or not known to be pregnant), and cardiopulmonary resuscitation (in 24 h before CCU admission). The outcomes of interest for the critical care cohort were duration of organ support (respiratory, cardiovascular, renal, neurological, and liver) in critical care, duration of critical care, and mortality at the end of critical care.

We used the primary care cohort to determine the association of lineage B.1.1.7 with admission to a CCU after a positive test and with the risk of 28-day mortality. We used the critical care cohort to determine the association of lineage B.1.1.7 with duration of organ support in critical care, duration of critical care, and mortality at the end of critical care. The patient cohorts are reported according to the RECORD guidelines.^16^

### Statistical analysis

We used flexible parametric survival models (Royston-Parmar model) to estimate the hazard ratio (HR) for 28-day mortality and admission to CCU comparing patients with lineage B.1.1.7 with those without in the primary care cohort, and for mortality at the end of critical care in the critical care cohort. In all the models, we chose degrees of freedom to minimise the Akaike information criterion, and we tested possible interactions between lineage B.1.1.7 and age, sex, and ethnicity using the Wald test. When the proportional hazard assumption was not met, a time varying HR was modelled.

For each cohort, we accounted for missing data by using multiple imputation by chained equations, which generated five imputed datasets. The imputation model included age, sex, the outcome of interest (28-day mortality, CCU admission, or critical care mortality), and all confounding and mediating variables. We fitted Royston-Parmar models within each imputed dataset and combined them in accordance with Rubin's rules.

A post-hoc power calculation showed that there is 80% power at the 0·05 significance level to detect an HR higher than 1·04 or lower than 0·96 for admission to CCU and higher than 1·09 or lower than 0·91 for mortality at the end of critical care in the lineage B.1.1.7 group.

For estimating the HR of 28-day mortality and CCU admission in the primary care cohort, the models were adjusted for patients' demographics (age, sex, deprivation index, geographical region, ethnicity, housing category [care home, homeless, or neither], BMI, and smoking status) and comorbidities (asthma, chronic obstructive pulmonary disease, type 1 or 2 diabetes, and hypertension). Age was modelled by use of a restricted cubic spline with four degrees of freedom. A random effect frailty term was included to account for similarities among patients registered in the same primary care practice.

The model used to estimate the association between lineage B.1.1.7 and risk of mortality at the end of critical care was adjusted by age, sex, ethnicity, BMI, deprivation index, severe comorbidities, dependency before admission to acute hospital, and geographical region. We included a random frailty term to account for similarities among patients registered in the same CCU. Including the age variable as a linear term rather than a restricted cubic spline did not change the results, and thus the linear term was included in the final model.

Given the timing of emergence of B.1.1.7, some time-dependent factors exist that might be seen as confounders on the association between B.1.1.7 and severe outcomes. B.1.1.7 appeared as the number of cases were increased—even facilitating this rise, given its higher transmissibility.^3^ The overwhelming of the health system, poorer care in very stretched units, and lower CCU bed availability are some of the factors that would increase mortality, and they might have increased the risk of mortality associated with lineage B.1.1.7 if not controlled for. To address these factors, we introduced in all models the 2-week period in which the test was done (overall 28-day mortality and CCU admission) or in which the patients had received critical care (critical care mortality).

The start time for the mortality and admission to CCU analyses using the primary care cohort was the date of a positive test. Patients were followed up for 28 days when exploring the relative risk of mortality between the B.1.1.7 and non-B.1.1.7 groups, and followed up to Feb 7, 2021, when investigating the relative risk of admission to CCU. Individuals who did not receive critical care up to Feb 7 (for CCU admission analysis) or who did not die within 28 days (for mortality analysis) were censored on Feb 7 (for CCU admission analysis) or 28 days after the date of a positive SARS-CoV-2 test (for mortality analysis). Patients who died during the follow-up period before receiving critical care or due to non-COVID-19 causes were censored at their date of death. For the analysis of mortality at the end of critical care in the critical care cohort, the start date was the date of their CCU admission. Patients were censored after 28 days, and those who survived were censored on their date of discharge from critical care. The follow-up period ended on Feb 7, 2021.

Patients with lineage B.1.1.7 became the majority of patients with COVID-19 at the end of the study period (appendix p 2). This caused patients with B.1.1.7 to have a shorter follow-up time and possibly to have their outcome not completed by the end of the study period. Therefore, in the critical care analysis, we did a sensitivity analysis including only patients who had completed their critical care outcomes (death or survival at discharge). All analyses were re-run including only the complete case dataset as an additional sensitivity analysis.

The outcome of 28-day mortality was chosen because it was available for all patients irrespective of date of admission, by contrast with information on types and duration of organ support, for which there was more availability in patients who were admitted earlier on in the study period. Therefore, to compare the clinical characteristics including organ support of patients in the B.1.1.7 group and in the non-B.1.1.7 group, we restricted our reporting to a matched cohort of patients, derived from the critical care cohort. Each patient critically ill with B.1.1.7 was matched with a patient critically ill without B.1.1.7 admitted to the same unit. Only pairs of patients who were admitted within 3 days of each other were used in the analysis. If a patient with lineage B.1.1.7 was matched with more than one patient without B.1.1.7, only one pair was randomly selected. Stata/MP, version 16.1, was used for these analyses.

### Role of the funding source

The funder of the study had no role in study design, data collection, data analysis, data interpretation, or writing of the report.

## Results

**Figure 1:**
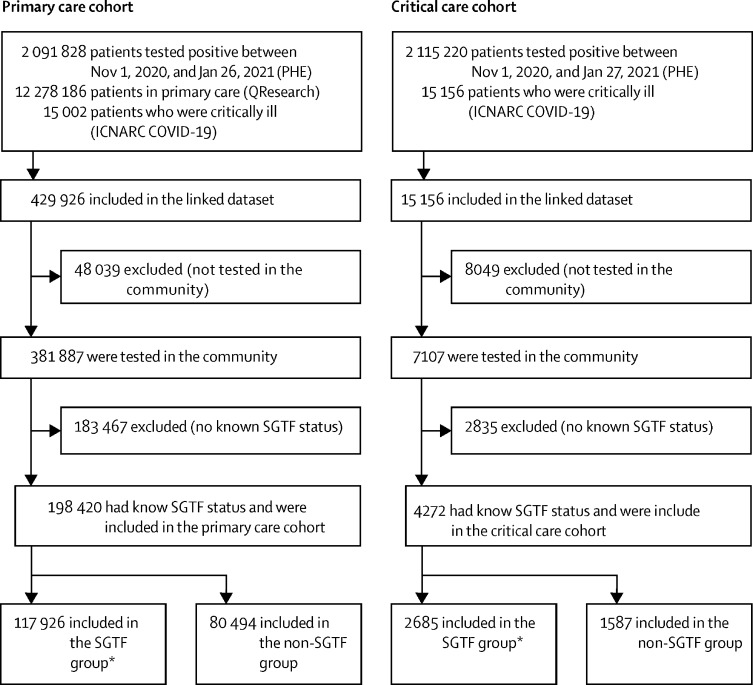
Flowchart of the primary care cohort and the critical care cohort ICNARC=Intensive Care National Audit & Research Centre. PHE=Public Health England. SGTF=S-gene molecular diagnostic assay failure. *SGTF is used as a proxy for lineage B.1.1.7.

Between Nov 1, 2020, and Jan 26, 2021, 2 091 828 patients had a COVID-19 RT-PCR test result and were recorded in the PHE database (figure 1). These patients were linked with the 12 278 186 patients registered with participating primary care practices in QResearch. This resulted in 429 926 primary care patients who had a positive COVID-19 RT-PCR test in the study inclusion period, of whom 381 887 had been tested in the community. SGTF status (as a proxy for B.1.1.7) was identifiable for 198 420 (51·9%) patients, who constituted the primary care cohort (figure 1). Demographics of patients tested in the community by whether SGTF status was available are shown in the appendix (pp 8–9). In the primary care cohort, 899 (0·4%) of 198 420 patients had died of COVID-19 within 28 days. Additionally, this cohort was linked to the ICNARC COVID-19 study database, identifying 836 (0·4%) patients who were admitted for critical care before Feb 7, 2021. The community test date was after the recorded date of death in 13 patients in the primary care cohort and in none of the patients in the critical care cohort.

Of 198 420 patients for whom results were available, 117 926 (59·4%) had SGTF and 80 494 (40·6%) had non-SGTF (figure 1). SGTF became increasingly dominant over the study period (appendix p 2). Patients in the SGTF group and the non-SGTF group had broadly similar characteristics, with some regional differences; however, the SGTF group had a lower proportion of patients aged 70 years or older (table 1). In the primary care cohort, data were missing for the variables BMI (24**·3**% of patients), smoking status (15·0%), deprivation quintile (0·7%), and ethnicity (20·1%; table 1).

**Table 1:** Demographics of primary care patients who tested positive in the community between Nov 1, 2020, and Jan 26, 2021 (primary care cohort), by lineage status

		**Non-SGTF (n=80 494)**	**SGTF (n=117 926)**	**Full cohort (n=198 420)**
Admitted to CCU		271 (0·3%)	565 (0·5%)	836 (0·4%)
Deaths within 28 days		334 (0·4%)	565 (0·5%)	899 (0·5%)
Sex				
	Female	42 976 (53·4%)	61 679 (52·3%)	104 655 (52·7%)
	Male	37 518 (46·6%)	56 247 (47·7%)	93 765 (47·3%)
Mean age, years		38·0 (18·1)	37·4 (17·6)	37·7 (17·8)
Age categories, years				
	18–29	29 122 (36·2%)	42 000 (35·6%)	71 122 (35·8%)
	30–39	15 412 (19·1%)	24 012 (20·4%)	39 424 (19·9%)
	40–49	13 255 (16·5%)	20 622 (17·5%)	33 877 (17·1%)
	50–59	12 604 (15·7%)	18 342 (15·6%)	30 946 (15·6%)
	60–69	6318 (7·8%)	8662 (7·3%)	14 980 (7·5%)
	70–79	2510 (3·1%)	2994 (2·5%)	5504 (2·8%)
	80–89	1022 (1·3%)	1073 (0·9%)	2095 (1·1%)
	90–99	251 (0·3%)	221 (0·2%)	472 (0·2%)
Ethnicity				
	White	49 016 (60·9%)	68 907 (58·4%)	117 923 (59·4%)
	Indian	3046 (3·8%)	4900 (4·2%)	7946 (4·0%)
	Pakistani	4045 (5·0%)	4323 (3·7%)	8368 (4·2%)
	Bangladeshi	2008 (2·5%)	3549 (3·0%)	5557 (2·8%)
	Other Asian	1526 (1·9%)	2958 (2·5%)	4484 (2·3%)
	Caribbean	400 (0·5%)	1339 (1·1%)	1739 (0·9%)
	Black African	1295 (1·6%)	2803 (2·4%)	4098 (2·1%)
	Chinese	159 (0·2%)	373 (0·3%)	532 (0·3%)
	Other ethnic group	2647 (3·3%)	5162 (4·4%)	7809 (3·9%)
	Not recorded	16 352 (20·3%)	23 612 (20·0%)	39 964 (20·1%)
Date of positive test				
	Nov 1–14, 2020	26 160 (32·5%)	1784 (1·5%)	27 944 (14·1%)
	Nov 15–28, 2020	16 509 (20·5%)	2879 (2·4%)	19 388 (9·8%)
	Nov 29 to Dec 12, 2020	10 990 (13·7%)	7704 (6·5%)	18 694 (9·4%)
	Dec 13–26, 2020	10 847 (13·5%)	23 700 (20·1%)	34 547 (17·4%)
	Dec 27, 2020, to Jan 10, 2021	11 368 (14·1%)	46 921 (39·8%)	58 289 (29·4%)
	Jan 11–26, 2021	4620 (5·7%)	34 938 (29·6%)	39 558 (19·9%)
Household size				
	1 person	21 580 (26·8%)	30 432 (25·8%)	52 012 (26·2%)
	2 people	16 618 (20·6%)	23 358 (19·8%)	39 976 (20·1%)
	3–5 people	36 192 (45·0%)	55 186 (46·8%)	91 378 (46·1%)
	≥6 people	6104 (7·6%)	8950 (7·6%)	15 054 (7·6%)
House type				
	Neither	80 219 (99·7%)	117 549 (99·7%)	197 768 (99·7%)
	Care home	226 (0·3%)	271 (0·2%)	497 (0·3%)
	Homeless	49 (0·1%)	106 (0·1%)	155 (0·1%)
BMI				
	<25	43 759 (54·4%)	64 816 (55·0%)	108 575 (54·7%)
	25 to <30	10 654 (13·2%)	14 706 (12·5%)	25 360 (12·8%)
	30 to <40	4528 (5·6%)	6007 (5·1%)	10 535 (5·3%)
	≥40	2540 (3·2%)	3151 (2·7%)	5691 (2·9%)
	Not recorded	19 013 (23·6%)	29 246 (24·8%)	48 259 (24·3%)
Smoking status				
	Non-smoker	46 182 (57·4%)	65 447 (55·5%)	111 629 (56·3%)
	Ex-smoker	14 262 (17·7%)	20 642 (17·5%)	34 904 (17·6%)
	Light smoker	6455 (8·0%)	11 276 (9·6%)	17 731 (8·9%)
	Moderate smoker	1222 (1·5%)	2050 (1·7%)	3272 (1·6%)
	Heavy smoker	429 (0·5%)	702 (0·6%)	1131 (0·6%)
	Not recorded	11 944 (14·8%)	17 809 (15·1%)	29 753 (15·0%)
Geographical region in England				
	East Midlands	1529 (1·9%)	1305 (1·1%)	2834 (1·4%)
	East of England	1428 (1·8%)	3912 (3·3%)	5340 (2·7%)
	London	13 302 (16·5%)	37 342 (31·7%)	50 644 (25·5%)
	North East	4066 (5·1%)	2194 (1·9%)	6260 (3·2%)
	North West	26 808 (33·3%)	22 416 (19·0%)	49 224 (24·8%)
	South Central	6754 (8·4%)	15 073 (12·8%)	21 827 (11·0%)
	South East	3609 (4·5%)	17 945 (15·2%)	21 554 (10·9%)
	South West	3966 (4·9%)	3893 (3·3%)	7859 (4·0%)
	West Midlands	14 235 (17·7%)	12 140 (10·3%)	26 375 (13·3%)
	Yorkshire and Humber	4797 (6·0%)	1706 (1·4%)	6503 (3·3%)
Deprivation quintile				
	1 (least deprived)	17 606 (21·9%)	22 650 (19·2%)	40 256 (20·3%)
	2	17 507 (21·7%)	25 312 (21·5%)	42 819 (21·6%)
	3	17 128 (21·3%)	25 327 (21·5%)	42 455 (21·4%)
	4	16 054 (19·9%)	23 521 (19·9%)	39 575 (19·9%)
	5 (most deprived)	11 622 (14·4%)	20 321 (17·2%)	31 943 (16·1%)
	Not recorded	577 (0·7%)	795 (0·7%)	1372 (0·7%)
Comorbidities				
	Asthma	12 551 (15·6%)	17 241 (14·6%)	29 792 (15·0%)
	COPD	875 (1·1%)	998 (0·8%)	1873 (0·9%)
	Diabetes type 1	440 (0·5%)	618 (0·5%)	1058 (0·5%)
	Diabetes type 2	4101 (5·1%)	5188 (4·4%)	9289 (4·7%)
	Hypertension	8473 (10·5%)	11 163 (9·5%)	19 636 (9·9%)
	Parkinson	64 (0·1%)	82 (0·1%)	146 (0·1%)
	Epilepsy	872 (1·1%)	1278 (1·1%)	2150 (1·1%)
	Cerebral palsy	73 (0·1%)	107 (0·1%)	180 (0·1%)
	Motor neuron disease	<5 (<0·1%)	5 (<0·1%)	8 (<0·1%)
	Huntington's disease	<5 (<0·1%)	5 (<0·1%)	6 (<0·1%)
	Multiple sclerosis	115 (0·1%)	152 (0·1%)	267 (0·1%)
	Myasthenia	20 (<0·1%)	28 (<0·1%)	48 (<0·1%)
	Down syndrome	30 (<0·1%)	51 (<0·1%)	81 (<0·1%)
	Learning disabilities excluding Down	1343 (1·7%)	1798 (1·5%)	3141 (1·6%)

Data are n (%) or mean (SD). Mortality data is updated up to Feb 23, 2021, and CCU admission data up to Feb 7, 2021. Values lower than five are not disclosed for protection of patient confidentiality. BMI=body-mass index. CCU=critical care unit. COPD=chronic obstructive pulmonary disease. SGTF=S-gene molecular diagnostic assay failure.

**Figure 2:**
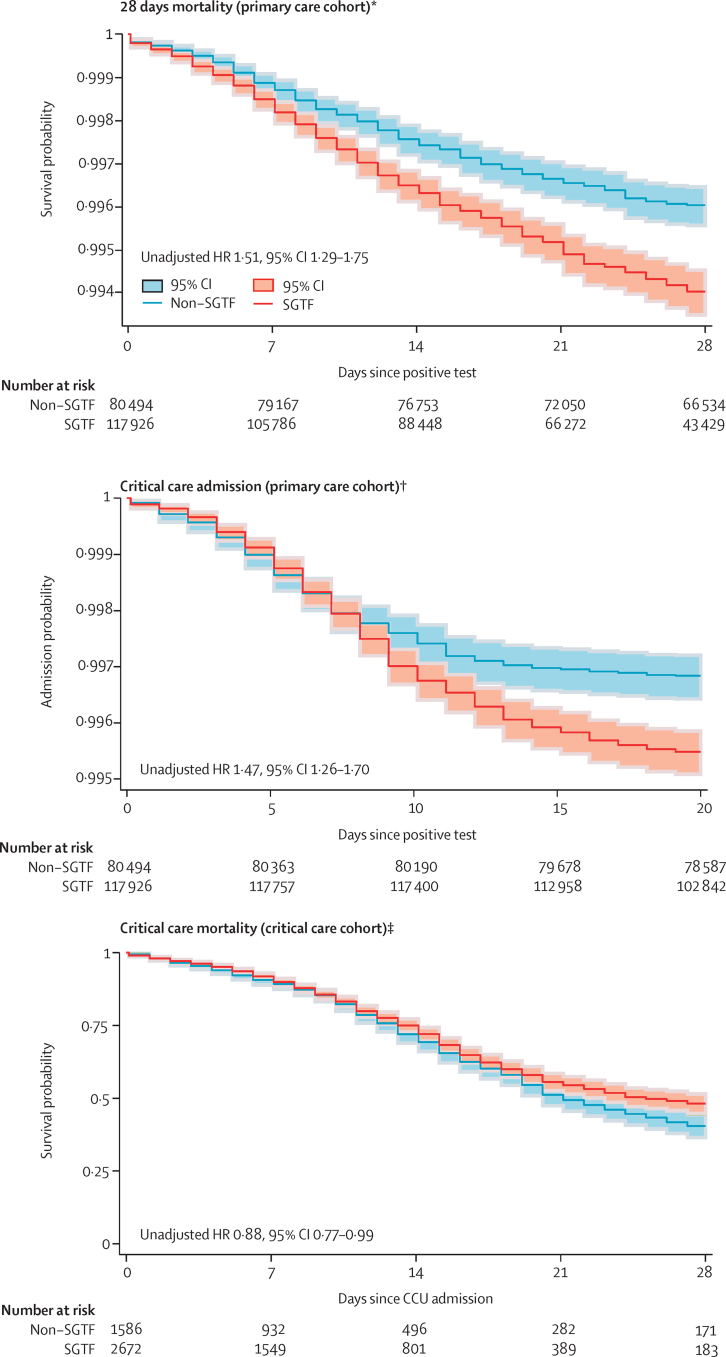
Risk of 28-days mortality and critical care admission in the primary care cohort, and mortality in the critical care cohort SGTF was used as a proxy for lineage B.1.1.7. INARC=Intensive Care National Audit & Research. HR=hazard ratio. PHE=Public Health England. SGTF=S-gene molecular diagnostic assay failure. *Unadjusted, complete case analysis for the period between Nov 1, 2020, and Feb 23, 2021; data sources: QReserach, INARC COVID−19 study, and PHE. †Unadjusted, complete case analysis for the period between Nov 1, 2020, and Jan 7, 2021; data sources: QReserach, INARC COVID−19 study, and PHE. ‡Analysis for the period between Nov 1, 2020, and Jan 27, 2021; data sources: ICNARC and PHE.

565 (0·5%) of 117 926 patients died in the SGTF group and 334 (0·4%) of 80 494 in the non-SGTF group. The weekly deaths by SGTF status over the study period are shown in the appendix (p 2). Figure 2 shows the Kaplan-Meier plot for risk of COVID-19 28-day mortality, by lineage, for the complete case analysis.

Unadjusted analysis indicated an increase in COVID-19 28-day mortality for patients in the SGTF group (unadjusted HR 1·51, 95% CI 1·29–1·75) compared with those in the non-SGTF group, which remained after adjustment (1·65, 1·36–2·01). We found no evidence of a significant interaction between lineage B.1.1.7 and ethnic group (p=0·75), sex (p=0·35), or age group (p=0·30; appendix pp 5–6). In total, 836 patients were admitted for critical care in the primary care cohort. Of these, 565 (0·5%) were in the SGTF group. The weekly CCU admissions by SGTF status over the study period are shown in the appendix (p 2). Figure 2 shows the Kaplan-Meier plot for risk of admission to critical care, by SGTF group, for the complete case analysis.

**Figure 3:**
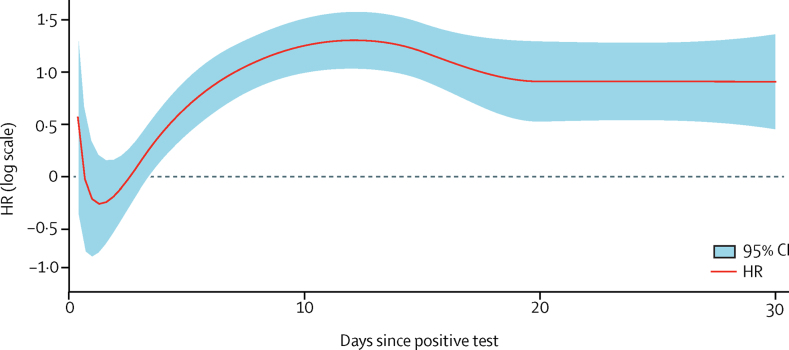
Estimated adjusted HR for critical care admission in the primary care cohort Adjusted, complete case analysis for the period between Nov 1, 2020, and Jan 26, 2021. Data sources used were QReserach, Intensive Care National Audit & Research COVID−19 study, and Public Health England. S-gene molecular diagnostic assay failure was used as a proxy for lineage B.1.1.7. HR=hazard ratio.

The risk of admission to critical care was higher in the SGTF group compared with the non-SGTF group in both unadjusted (HR 1·47, 95% CI 1·26–1·70) and adjusted (2·15, 1·75–2·65) analyses. However, the proportional hazard assumption was not met, so the time varying HR was estimated (figure 3). The time varying HR was 0·72 (0·40–1·26) 1 day after a positive test, 1·89 (1·41–2·53) 5 days after, 3·24 (2·41–4·36) 15 days after, and 2·41 (1·59–3·63) 20 days after. We found no evidence of a significant interaction between SGTF group and sex (p=0·95), ethnic group (p=0·49), or age group (p=0·23; appendix pp 5–6). Adjusting only for the date of positive test did not account for the increased risk of admission to CCU in the SGTF group versus the non-SGTF group (adjusted HR 1·37, 95% CI 1·14–1·65).

Between Nov 1, 2020, and Jan 27, 2021, 2 115 220 patients had a positive COVID-19 RT-PCR test and were recorded in PHE. Of these, 15 156 were admitted to CCU in the ICNARC COVID-19 study before Feb 7, 2021. Of these 15 156 patients in CCU, 7107 had a positive COVID-19 RT-PCR test done in the community (not hospital) before CCU admission. SGTF status (as a proxy for lineage B.1.1.7) was identifiable for 4272 (60·0 %) patients (figure 1), who were included in the critical care cohort. Of the 4272 patients in the critical care cohort, 2685 (62·8%) had SGTF and 1587 (37·2%) had non-SGTF (figure 1). Figure 2 shows the Kaplan-Meier plot for risk of admission for critical care, by lineage, for the complete case analysis.

Patients in the SGTF group tended to be marginally younger and be in the lowest BMI category than those of the non-SGTF group (table 2). Compared with the non-SGTF group, acute severity of illness, measured by the Acute Physiology And Chronic Health Evaluation II (also known as APACHE II) score, tended to be lower in the SGTF group, but the proportion receiving invasive mechanical ventilation within the first 24 h of critical care was higher in the SGTF group. 1851 (68·9%) patients in the SGTF group had completed their CCU stay at the point of analysis, compared with 1455 (91·7%) patients in the non-SGTF group (additional demographic characteristics are shown in appendix pp 8–9). In the critical care cohort, data were missing for the variables ethnicity (9·2%) and dependency status before admission (9·4%; table 2).

**Table 2:** Demographic characteristics and medical characteristics and indicators of acute severity observed for patients who were critically ill and tested positive in the community between Nov 1, 2020, and Feb 7, 2021 (critical care cohort), by lineage

		**Non-SGTF (n=1587)**	**SGTF (n=2685)**	**Full cohort (n=4272)**
Mean age, years		59·2 (12·8)	58·1 (12·2)	58·5 (12·5)
Sex				
	Female	521 (32·8%)	908 (33·8%)	1429 (33·5%)
	Male	1066 (67·2%)	1777 (66·2%)	2843 (66·5%)
Ethnicity				
	White	1149 (72·4%)	1754 (65·3%)	2903 (68·0%)
	Indian	63 (4·0%)	106 (3·9%)	169 (4·0%)
	Pakistani	96 (6·0%)	128 (4·8%)	224 (5·2%)
	Bangladeshi	23 (1·4%)	51 (1·9%)	74 (1·7%)
	Other Asian	62 (3·9%)	120 (4·5%)	182 (4·3%)
	Caribbean	13 (0·8%)	35 (1·3%)	48 (1·1%)
	Black African	13 (0·8%)	47 (1·8%)	60 (1·4%)
	Chinese	7 (0·4%)	10 (0·4%)	17 (0·4%)
	Other ethnic group	63 (4·0%)	141 (5·3%)	204 (4·8%)
	Not recorded	98 (6·2%)	293 (10·9%)	391 (9·2%)
Previous length of hospital stay, days				
	Mean	2·5 (12·9; n=1580)	3·0 (15·9; n=2640)	2·8 (14·9; n=4220)
	Median	1 (0–3; n=1580)	1 (0–3; n=2640)	1 (0–3; n=4220)
Dependency before admission to acute hospital care				
	Able to live without assistance in daily activities	1378 (86·8%)	2189 (81·5%)	3567 (83·5%)
	Some assistance in daily activities	133 (8·4%)	161 (6·0%)	294 (6·9%)
	Total assistance with all daily activities	<5 (<0·1%)	<5 (<0·1%)	9 (0·2%)
	Not recorded	72 (4·5%)	330 (12·3%)	402 (9·4%)
Severe comorbidities				
	Cardiovascular	<5 (<0·1%)	>10	17 (0·4%)
	Respiratory	11 (0·7%)	21 (0·8%)	32 (0·7%)
	Renal*	9 (0·6%)	10 (0·4%)	19 (0·4%)
	Liver	>10	<5 (<0·1%)	9 (0·2%)
	Metastatic disease	5 (0·3%)	5 (0·2%)	10 (0·2%)
	Haematological malignancy	13 (0·8%)	10 (0·4%)	23 (0·5%)
	Immunocompromised	32 (2·0%)	41 (1·5%)	73 (1·7%)
BMI				
	<25	376 (23·7%)	851 (31·7%)	1227 (28·7%)
	25 to <30	439 (27·7%)	649 (24·2%)	1088 (25·5%)
	30 to <40	587 (37·0%)	870 (32·4%)	1457 (34·1%)
	≥40	185 (11·7%)	315 (11·7%)	500 (11·7%)
CPR within preceding 21 h				
	In the community	12 (0·8%)	8 (0·3%)	20 (0·5%)
	In the hospital	10 (0·6%)	20 (0·7%)	30 (0·7%)
	No	1526 (96·2%)	2469 (92·0%)	3995 (93·5%)
	Not recorded	39 (2·5%)	188 (7·0%)	227 (5·3%)
Currently or recently pregnant				
	Currently pregnant	7 (0·4%)	17 (0·6%)	24 (0·6%)
	Recently pregnant (within 6 weeks)	5 (0·3%)	19 (0·7%)	24 (0·6%)
	Not known to be pregnant	1575 (99·2%)	2649 (98·7%)	4224 (98·9%)
Invasively ventilated within first 24 h				
	No	1131 (71·3%)	1469 (54·7%)	2600 (60·9%)
	Yes	372 (23·4%)	724 (27·0%)	1096 (25·7%)
	Not recorded	84 (5·3%)	492 (18·3%)	576 (13·5%)
APACHE II score				
	Mean	13·9 (5·1; n=1527)	13·0 (4·9; n=2292)	13·3 (5·0; n=3819)
	Median	13 (11–16; n=1527)	13 (10–16; n=2292)	13 (10–16; n=3819)
PaO_2_/FiO_2_ ratio median		13·2 (10·0–18·2; n=1416)	13·6 (9·8–18·0; n=2056)	13·3 (9·8–18·2; n=3472)
PaO_2_/FiO_2_ ratio value				
	<13·3 kPa (<100 mm Hg)	718 (45·2%)	1022 (38·1%)	1740 (40·7%)
	13·3–26·6 kPa (100–200 mm Hg)	585 (36·9%)	873 (32·5%)	1458 (34·1%)
	>26·6 kPa (>200 mm Hg)	113 (7·1%)	161 (6·0%)	274 (6·4%)
	Not recorded	718 (45·2%)	1022 (38·1%)	1740 (40·7%)
	FiO_2_ [N = 1416/2056] median	0·60 (0·45–0·75; n=1416)	0·60 (0·45–0·80; n=2056)	0·60 (0·45–0·80; n=3472)

Data are n (%), mean (SD), or median (IQR). Values lower than five and those denoted as “>10” are not disclosed for protection of patient confidentiality. APACHE II=Acute Physiology And Chronic Health Evaluation II. BMI=body-mass index. CPR=cardiopulmonary resuscitation. FiO_2_=fractional inspired oxygen. PaO_2_=arterial oxygen partial pressure. SGTF=S-gene molecular diagnostic assay failure.

* Chronic, irreversible renal disease and being dialysis-dependent before critical care unit admission.

We observed a lower risk of mortality in critical care in the SGTF group, in the unadjusted analysis (HR 0·88, 0·77–0·99). After adjusting for additional confounders and date of admission, critical care mortality did not differ significantly between the SGTF and non-SGTF groups (0·91, 0·76–1·09). We found no evidence of a significant interaction between SGTF and ethnic group (p=0·34), age group (p=0·53), or sex (p=0·95).

In the critical care cohort, 2393 (56%) of patients had a follow-up of at least 28 days, and of these, 1315 (54·9%) were in the non-B.1.1.7 group. These patients were included in the sensitivity analyses, including only patients already discharged from critical care (alive or dead), the findings of which were consistent with the main analysis (characteristics of the critical care cohort restricted to those who had completed their critical care stay are summarised in appendix pp 8–9). In the primary care cohort, a sensitivity analysis for 28-day mortality was not required because all patients had complete follow-up to 28 days. Similarly, for CCU admission outcome, 185 127 (93·3%) of 198 420 patients in the primary care cohort had a potential follow-up period of 20 days and, given that 90% of patients who needed critical care were admitted within 16 days of the positive test, a sensitivity analysis did not seem necessary.

Complete case analyses were all consistent with the imputed analyses.

Overall, in the matched cohort of patients who were critically ill, organ support receipt was similar between the two groups (table 3). Demographic and clinical characteristics of patients in the matched cohort are shown in the appendix (pp 10–11).

**Table 3:** Critical care outcomes for the matched cohort of patients who were critically ill with a positive test in the community between Nov 1, 2020, and Feb 7, 2021, by lineage

	**Non-SGTF (n=1031)**	**SGTF (n=1031)**	**Full cohort (n=2062)**
**Outcome at end of critical care**			
Discharged	553 (53·6%)	491 (47·6%)	1044 (50·6%)
Died	289 (28·0%)	262 (25·4%)	551 (26·7%)
Still receiving critical care	189 (18·3%)	278 (30·0%)	467 (22·6%)
**Duration of critical care, days**			
Discharged patients	5 (4–8)	5 (3–9)	5 (3–9)
Deaths	11 (4–16)	11 (6–16)	11 (5–16)
Organ support*			
No respiratory support	51 (6·1%)	30 (4·0%)	81 (5·1%)
Advanced respiratory support	401 (47·6%)	340 (45·2%)	741 (46·5%)
Basic respiratory support	672 (79·8%)	635 (84·3%)	1307 (81·9%)
No cardiovascular support	78 (9·3%)	56 (7·4%)	134 (8·4%)
Advanced cardiovascular support	189 (22·4%)	135 (17·9%)	324 (20·3%)
Basic cardiovascular support	750 (89·1%)	688 (91·4%)	1438 (90·2%)
Renal support	142 (16·9%)	101 (13·4%)	243 (15·2%)
Liver support	31 (3·7%)	39 (5·2%)	70 (4·4%)
Neurological support	53 (6·3%)	58 (7·7%)	111 (7·0%)
Duration of organ support, days*			
Advanced respiratory support	8 (5–13)	9 (5–14)	8 (5–14)
Total (advanced plus basic) respiratory support	7 (7–11)	7 (4–13)	7 (4–12)
Advanced cardiovascular support	3 (1–4)	2 (1–5)	3 (1–4)
Total (advanced plus basic) cardiovascular support	7 (5–12)	7 (4–13)	7 (4–14)
Renal support	3 (3–6)	5 (3–9)	4 (3–7)

Data are n (%) or median (IQR). Each patient in the SGTF group was matched with a patient in the non-SGTF group admitted to the same CCU unit. Only patients who were admitted within 3 days of each other were allowed to be matched. For each patient with SGTF status, only one matched set was randomly selected. The matched cohort consisted of 2062 patients (761 in the SGTF group and 1031 in the non-SGTF group). CCU=critical care unit. SGTF=S-gene molecular diagnostic assay failure.

* Among patients who have been discharged or died.

## Discussion

To our knowledge, this is the first study to report risks of admission to CCU and clinical outcomes among patients admitted to critical care, comparing patients positive for lineage B.1.1.7 with those positive for non-B.1.1.7 SARS-CoV-2. Lineage B.1.1.7 became dominant over the study period. Using SGTF results as a proxy for lineage B.1.1.7, we found a substantially increased risk of overall COVID-19 28-day mortality and of admission to CCU associated with B.1.1.7, but no difference in risk of critical care mortality or organ support receipt for patients in CCU.

Previous studies have shown a higher risk of overall mortality associated with the B.1.1.7 lineage^4^, ^5^, ^6^, ^7^, ^8^, ^9^ and no association with mortality in patients admitted to hospital.^10^ Our study is in agreement, showing that, once the patient is admitted to CCU, outcomes are similar between those in the B.1.1.7 and non-B.1.1.7 groups. This finding might result from patients in either group having similar acute severity of illness at the point of admission.

We found a 65% higher risk of COVID-19 28-day mortality for patients with lineage B.1.1.7 compared with those in the non-B.1.1.7 group. Our study showed that the highly prevalent B.1.1.7 infects a population similar to that infected with non-B.1.1.7 SARS-CoV-2, albeit with fewer patients aged 70 years or older having a positive community test for lineage B.1.1.7. Whether this is a true difference in who the lineage infects or a difference in exposure or in testing remains unclear. An advantage of our study is that the primary care cohort had previous recording of a wide range of exposures and comorbidities. This allowed us to adjust the analysis, controlling for many important potential confounders. To attenuate the effect of time-dependent confounders, we adjusted for an indicator of the 2-week period in which the patient tested positive or started receiving critical care.

Infection with lineage B.1.1.7 was associated with a doubling of the risk of admission to CCU compared with infection with non-B.1.1.7 SARS-CoV-2. Although the increased infectivity of B.1.1.7 has been reported,^2^, ^3^ we are not aware of previous work examining the risk of CCU admission. Adjusting only for the date of positive test did not explain this increased risk of CCU admission, suggesting that the effect is not explained by time-dependent factors such as CCU bed availability. Although this finding raises concerns about future capacity planning, it should be interpreted with some caution. For example, in our study population, lineage B.1.1.7 appeared to be more prevalent in younger people than in older individuals, and we do not know, with the data available, whether this will remain the case as lineage B.1.1.7 spreads. The large preponderance of men admitted to CCU, despite fewer men having a positive COVID-19 RT-PCR test, has been previously reported.^17^ Our work showed that this pattern remains with lineage B.1.1.7. Both findings are similar to those reported for UK data for all CCU admissions, where men were admitted in higher proportions than women.^17^

Our study has some important strengths. It uses established, complete, and validated data sources that are either the national databases for England (PHE and ICNARC COVID-19 study) or a very large representative sample (QResearch).^14^, ^15^, ^18^, ^19^ Outcome data, for both patient cohorts, were complete and missing data occurred only in some predictor variables. Multiple imputation agreed with complete case analyses in both cohorts. The use of the national register also minimised the risk of misclassification bias; however, misclassification bias might still have occurred. A substantial limitation of the data available for our work is that the determination of SGTF status, as a proxy for B.1.1.7, was only possible in just over 50% of patients with a community COVID-19 RT-PCR positive test. Patients who died after being tested in the community and care-home residents were less likely to have SGTF status identified (appendix pp 3–4). A potential limitation of this study is that our analysis was restricted to positive tests with Ct of 30 or lower, in line with national guidance.^5^ Selection bias might be introduced if the two lineage groups show a striking difference in the Ct values at the date of the positive test. However, previous work^4^ suggests that adjusting for Ct values does not affect mortality outcomes.

To minimise the risks of changing eligibility over time, we restricted our analysis to an 11-week period where lineage B.1.1.7 had become noteworthy. We used established measures, including critical care mortality and measures of critical care severity, such as duration of CCU stay and receipt of basic and advanced organ support. Our data are very timely, being reported during the third pandemic wave in England. We could not observe patients who died before being tested. If the early risk of death differs between lineages, this could introduce bias. As with all observational studies, our study remains subject to unmeasured confounding, particularly because we are investigating the effects of new virus variants during a pandemic, where classification errors can occur.

Throughout this paper, we have compared lineage B.1.1.7 with non-B.1.1.7 SARS-CoV-2 by use of the SGTF status as proxy for the lineage. It should be acknowledged that the non-SGTF group might include other lineages, such as 501Y.V2 and B.1.351; however, given the prevalence of the different lineages in England at the time of the study, it seems likely that most non-B.1.1.7 cases represent the original virus.

Our study shows increased risk of COVID-19 28-day mortality and risk of CCU admission for patients who tested positive for lineage B.1.1.7. Combined with evidence of increased infectivity, our findings emphasise the importance of measures to control exposure to and infection with COVID-19.

## Data sharing

To guarantee the confidentiality of personal and health information, only the authors have had access to the data during the study in accordance with the relevant licence agreements. Access to the QResearch data is according to the information on the QResearch website. The open source software allows use without charge.

## Competing interests

JH-C reports receiving grants from the Wellcome trust, Health Data Research-UK, National Institute for Health Research (NIHR) Biomedical Research Centre (Oxford), John Fell Oxford University Press Research Fund, Cancer Research UK, and Oxford Wellcome Institutional Strategic Support Fund; being a member of SAGE subgroups on ethnicity and chair of the NERVTAG risk stratification subgroup; being an unpaid director of QResearch; and being a founder and former director of ClinRisk, outside the submitted work. PST reports previous consultations with AstraZeneca and Duke-National University of Singapore, outside the submitted work. PW was Chief Medical Officer for Sensyne Health, his department received research funding from Sensyne Health, and he holds shares in the company; and he received grant funding from the National Institute for Health Research and Wellcome Trust.
