# Supplementary material for: Mortality and critical care unit admission associated with the SARS-CoV-2 lineage B.1.1.7 in England: an observational cohort study
Source: Lancet Infect Dis. 2021 Nov;21(11):1518–28. doi: 10.1016/S1473-3099(21)00318-2 (PMC8219489; doi:10.1016/S1473-3099(21)00318-2)
Supplement: Supplementary appendix [file mmc1.pdf]

THE LANCET
Infectious Diseases

Supplementary appendix

This appendix formed part of the original submission and has been peer reviewed. We post it as supplied by the authors.

Supplement to: Patone M, ThomasK, Hatch R, et al. Mortality and critical care unit admission associated with the SARS-CoV-2 lineage B.1.1.7 in England: an observational cohort study. *Lancet Infect Dis* 2021; published online June 22. https://doi.org/10.1016/S1473-3099(21)00318-2.

Page content (appendix)

Page 2: (Top-left) Weekly positive tests by variant from 1st November to 26th January 2021, (top-right) weekly deaths by variant from 1st November to 23rd February 2021, (bottom-left) weekly critical care admissions per variant from 1st November to 7th February 2021, (bottom-right) weekly deaths at the end of critical care by variant from 1st November2020 to 7th February 2021. SGTF status was used as a proxy for VOC B.1.1.7.

Page 3: Demographics characteristics of primary care patients tested positive in hospital and community settings between 1st November 2020 and 26th January 2021 and of those with SGTF status known or not.

Page 5: Value of p-value for testing the interaction between sex, age and ethnicity with the SGTF status in each model.

Page 7: Critical care outcomes observed in the critical care cohort, by variant.

Page 8: Demographic, medical characteristics and indicators of acute severity observed for patients in the critical care cohort who have completed their critical care outcome (discharged alive or dead).

Page 10: Demographic, medical characteristics and indicators of acute severity observed in patients of the critical care-cohort after matching.

Page 12: Demographics characteristics of primary care patients tested positive in hospital and community settings between 1st November 2020 and 26th January 2021 by months.

Page 15: Counts of deaths by region compared with the ones observed in the primary care cohort. The counts of deaths in England were taken from https://coronavirus.data.gov.uk/details/deaths. All regions were covered in the primary care cohort for both the SGTF and non-SGTF

Appendix part 1: (Top-left) Weekly positive tests by variant from 1st November to 26th January 2021, (top-right) weekly deaths by variant from 1st November to 23rd February 2021, (bottom-left) weekly critical care admissions per variant from 1st November to 7th February 2021, (bottom-right) weekly deaths at the end of critical care by variant from 1st November2020 to 7th February 2021. SGTF status was used as a proxy for VOC B.1.1.7.

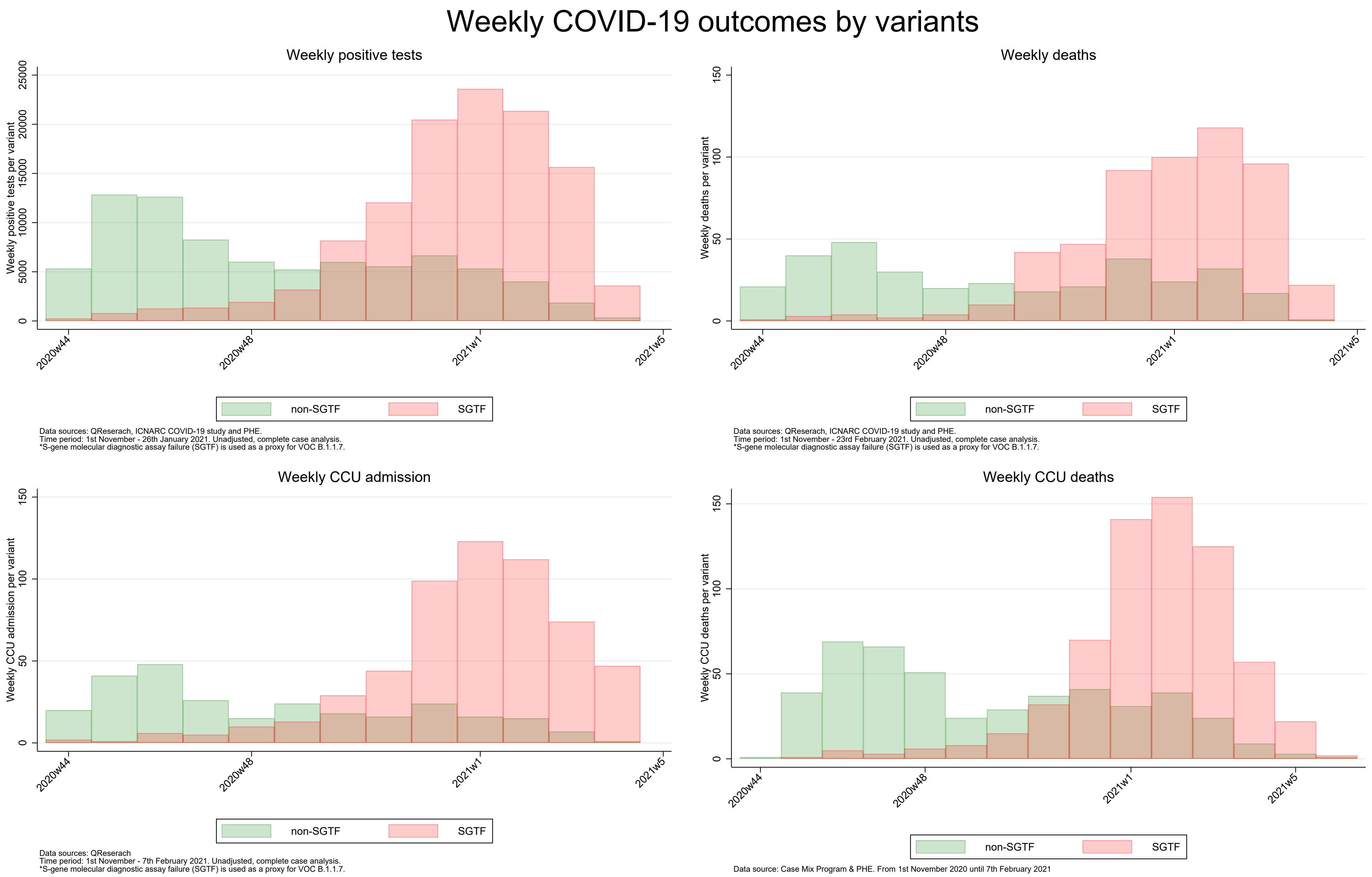

Appendix, part 2: Demographics characteristics of primary care patients tested positive in hospital and community settings between 1st November 2020 and 26th January 2021 and of those with SGTF status known or not.

	**Hospital settings**	**Community settings**	**SGTF status unknown (community test only)**	**SGTF status known (community test only)**
	Cols% (counts)	*Cols% (counts)*	*Cols% (counts)*	*Cols% (counts)*
**Total number of patients**	48039	381887	183467	198420
**Mean age (SD)**	56.7 (23.5)	38.3 (18.8)	39.1 (19.8)	37.7 (17.8)
**Deaths**	15.8 (7606)	0.9 (3493)	1.4 (2594)	0.5 (899)
**CCU admitted**	3.4 (1629)	0.3 (1248)	0.3 (536)	0.4 (836)
**SGTF**	0	30.9 (117926)		
**Sex**				
Female	55.0 (26408)	54.3 (207295)	55.9 (102640)	52.7 (104655)
Male	45.0 (21631)	45.7 (174592)	44.1 (80827)	47.3 (93765)
**Age groups**				
18-29	15.6 (7499)	35.6 (135991)	35.4 (64869)	35.8 (71122)
30-39	12.3 (5899)	19.5 (74447)	19.1 (35023)	19.9 (39424)
40-49	11.9 (5725)	16.6 (63563)	16.2 (29686)	17.1 (33877)
50-59	13.4 (6420)	15.1 (57743)	14.6 (26797)	15.6 (30946)
60-69	11.0 (5301)	7.5 (28539)	7.4 (13559)	7.5 (14980)
70-79	13.2 (6325)	3.0 (11627)	3.3 (6123)	2.8 (5504)
80-99	22.6 (10870)	2.6 (9977)	4.0 (7410)	1.3 (2567)
**Ethnicity**				
White	61.8 (29685)	58.7 (224187)	57.9 (106264)	59.4 (117923)
Indian	3.3 (1596)	3.9 (14829)	3.8 (6883)	4.0 (7946)
Pakistani	2.5 (1189)	3.6 (13700)	2.9 (5332)	4.2 (8368)
Bangladeshi	2.0 (965)	3.4 (13077)	4.1 (7520)	2.8 (5557)
Other Asian	2.5 (1204)	2.4 (9135)	2.5 (4651)	2.3 (4484)
Caribbean	2.1 (1017)	1.2 (4451)	1.5 (2712)	0.9 (1739)
Black African	3.5 (1698)	2.5 (9692)	3.0 (5594)	2.1 (4098)
Chinese	0.3 (146)	0.3 (1215)	0.4 (683)	0.3 (532)
Other ethnic group	4.4 (2120)	4.5 (17224)	5.1 (9415)	3.9 (7809)
Not recorded	17.5 (8419)	19.5 (74377)	18.8 (34413)	20.1 (39964)
**House size**				
1 person	39.9 (19185)	26.3 (100579)	26.5 (48567)	26.2 (52012)
2 people	23.7 (11407)	19.9 (75912)	19.6 (35936)	20.1 (39976)
3-5 people	28.8 (13848)	45.2 (172476)	44.2 (81098)	46.1 (91378)
6-10 people or more	7.5 (3599)	8.6 (32920)	9.7 (17866)	7.6 (15054)
**Period of positive test**				
1 Nov to 14 Nov	24.6 (11822)	22.0 (84091)	24.3 (44533)	19.6 (39558)
15 Nov to 28 Nov	11.0 (5303)	11.1 (42337)	7.8 (14393)	14.1 (27944)
29 Nov to 12 Dec	9.7 (4675)	8.1 (30853)	6.2 (11465)	9.8 (19388)
13 Dec to 26 Dec	10.2 (4920)	8.0 (30438)	6.4 (11744)	9.4 (18694)
27 Dec to 10 Jan	18.0 (8639)	17.3 (65985)	17.1 (31438)	17.4 (34547)
11 Jan to 26 Jan	26.4 (12680)	33.6 (128183)	38.1 (69894)	29.4 (58289)
**House type**				
Neither	96.3 (46242)	98.8 (377466)	97.9 (179698)	99.7 (197768)
Carehome	3.4 (1643)	1.1 (4062)	1.9 (3565)	0.3 (497)
Homeless	0.3 (154)	0.1 (359)	0.1 (204)	0.1 (155)
**BMI**				
<25	59.9 (28768)	55.4 (211753)	56.2 (103178)	54.7 (108575)
25-30	15.9 (7621)	12.6 (48021)	12.4 (22661)	12.8 (25360)
30-40	7.3 (3518)	5.2 (19709)	5.0 (9174)	5.3 (10535)
>= 40	4.0 (1941)	2.8 (10586)	2.7 (4895)	2.9 (5691)
Not recorded	12.9 (6191)	24.0 (91818)	23.7 (43559)	24.3 (48259)

**Smoking status**				
Non smoker	56.7 (27255)	55.9 (213519)	55.5 (101890)	56.3 (111629)
Ex smoker	26.2 (12593)	17.4 (66378)	17.2 (31474)	17.6 (34904)
Light smoker	8.7 (4167)	9.5 (36284)	10.1 (18553)	8.9 (17731)
Moderate smoker	1.8 (846)	1.7 (6630)	1.8 (3358)	1.6 (3272)
Heavy smoker	1.0 (475)	0.6 (2316)	0.6 (1185)	0.6 (1131)
Not recorded	5.6 (2703)	14.9 (56760)	14.7 (27007)	15.0 (29753)
**Geographical region**				
East Midlands	1.5 (741)	1.8 (6847)	2.2 (4013)	1.4 (2834)
East of England	3.8 (1827)	3.4 (13163)	4.3 (7823)	2.7 (5340)
London	28.0 (13441)	33.1 (126460)	41.3 (75816)	25.5 (50644)
North East	2.5 (1222)	1.9 (7435)	0.6 (1175)	3.2 (6260)
North West	18.4 (8853)	16.2 (61757)	6.8 (12533)	24.8 (49224)
South Central	11.3 (5433)	10.9 (41494)	10.7 (19667)	11.0 (21827)
South East	12.3 (5932)	13.4 (51299)	16.2 (29745)	10.9 (21554)
South West	8.4 (4015)	6.5 (24910)	9.3 (17051)	4.0 (7859)
West Midlands	10.9 (5248)	10.3 (39273)	7.0 (12898)	13.3 (26375)
Yorkshire & Humber	2.8 (1327)	2.4 (9249)	1.5 (2746)	3.3 (6503)
**Deprivation quintile**				
1 (least deprived)	18.8 (9042)	18.7 (71332)	16.9 (31076)	20.3 (40256)
2	20.0 (9628)	20.9 (79745)	20.1 (36926)	21.6 (42819)
3	21.4 (10258)	21.0 (80043)	20.5 (37588)	21.4 (42455)
4	20.7 (9936)	20.1 (76918)	20.4 (37343)	19.9 (39575)
5 (most deprived)	18.6 (8921)	18.7 (71386)	21.5 (39443)	16.1 (31943)
Not recorded	0.5 (254)	0.6 (2463)	0.6 (1091)	0.7 (1372)
**Comorbidities**				
Asthma	15.2 (7304)	14.5 (55557)	14.0 (25765)	15.0 (29792)
COPD	7.0 (3340)	1.1 (4032)	1.2 (2159)	0.9 (1873)
Diabetes type 1	0.9 (423)	0.5 (2086)	0.6 (1028)	0.5 (1058)
Diabetes type 2	17.2 (8246)	5.0 (19272)	5.4 (9983)	4.7 (9289)
Hypertension	33.6 (16141)	10.7 (41046)	11.7 (21410)	9.9 (19636)
Parkinson	1.2 (568)	0.2 (612)	0.3 (466)	0.1 (146)
Epilepsy	2.3 (1105)	1.2 (4638)	1.4 (2488)	1.1 (2150)
Cerebral palsy	0.2 (109)	0.1 (437)	0.1 (257)	0.1 (180)
MND	0.0 (17)	0.0 (17)	0.0 (9)	0.0 (8)
Huntington	0.0 (10)	0.0 (40)	0.0 (34)	0.0 (6)
Multiple sclerosis	0.4 (178)	0.1 (570)	0.2 (303)	0.1 (267)
Myasthenia	0.1 (41)	0.0 (95)	0.0 (47)	0.0 (48)
Downs syndrome	0.1 (69)	0.1 (229)	0.1 (148)	0.0 (81)
Learning disabilities	3.5 (1676)	1.8 (6969)	2.1 (3828)	1.6 (3141)

Appendix, part 3: Value of p-value for testing the interaction between sex, age and ethnicity with the SGTF status in each model.

	**HR (95% CI)**
**28-days mortality (primary care cohort)**	
Age group & SGTF (Wald test, p-value = 0.30)	
< 50 (baseline)	-
>= 50 & < 60	0.39 (0.13, 1.12)
>= 60 & < 70	0.48 (0.18, 1.33)
>= 70 & < 80	0.45 (0.17, 1.18)
>= 80	0.37 (0.14, 0.98)
Sex & SGTF (Wald test, p-value = 0.35)	
Female (baseline)	-
Male	0.86 (0.62, 1.19)
Ethnic group & SGTF (Wald test, p-value = 0.75)	
White (baseline)	**-**
Indian	1.06 (0.51, 2.19)
Pakistani	0.83 (0.42, 1.63)
Bangladeshi	1.57 (0.44, 5.61)
Chinese	0.41 (0.04, 4.22)
Other Asian	1.61 (0.34, 7.55)
Black African and Caribbean	1.20 (0.40, 3.61)
Others	2.27 (0.79, 6.50)
**CCU admission (primary care cohort)**	
Age group & SGTF (Wald test, p-value = 0.23)	
< 18 (baseline)	**-**
>= 18 & < 30	**0.96 (0.37, 1.29)**
>= 30 & < 40	**1.30 (0.71, 2.37)**
>= 40 & < 50	**1.30 (0.44, 3.85)**
>= 50 & < 60	**1.14 (0.36, 3.49)**
>= 60 & < 70	**0.51 (0.13, 2.03)**
>= 70 & < 80	**1.78 (0.51, 6.28)**
>= 80	**2.09 (0.79, 5.52)**
Sex & SGTF (Wald test, p-value = 0.95)	
Female (baseline)	**-**
Male	**0.99 (0.71, 1.39)**
Ethnic group & SGTF (Wald test, p-value = 0.49)	
White (baseline)	
Indian	**0.69 (0.37, 1.29)**
Pakistani	1.30 (0.71, 2.37)
Bangladeshi	1.29 (0.43, 3.84)
Other Asian and Chinese	1.14 (0.37, 3.48)
Caribbean	0.51 (0.13, 2.03)
Black African	1.78 (0.51, 6.28)
Others	2.09 (0.79, 5.52)
**28-days mortality (critical care cohort)**	
Age group & SGTF (Wald test, p-value = 0.53)	
< 35 (baseline)	-
>= 34 & < 45	3.17 (0.89, 11.36)
>= 45 & < 65	1.23 (0.60, 2.53)
>= 65 & < 75	2.03 (0.98, 4.20)
>= 75 & < 85	4.25 (2.03, 8.93)
>= 85	8.87 (2.99, 26.32)

Sex & SGTF (Wald test, p-value = 0.95)	
Female (baseline)	-
Male	1.03 (0.83, 1.28)
Ethnic group & SGTF (Wald test, p-value = 0.95)	
White (baseline)	-
Indian	0.96 (0.54, 1.70)
Pakistani	0.69 (0.42, 1.09)
Bangladeshi	0.72 (0.29, 1.78)
Other Asian and Chinese	1.27 (0.69, 2.34)
Caribbean	0.37 (0.11, 1.15)
Black African	0.56 (0.21, 1.51)
Others	0.76 (0.39, 1.47)

Appendix, part 4: Critical care outcomes observed in the critical care cohort, by variant.

	**Non-SGTF** *Cols% (counts)*	**SGTF** *Cols% (counts)*	**Full Cohort** *Cols% (counts)*
**Outcome at end of critical care**			
Discharged	64.3 (936)	64.2 (1189)	64.3 (2125)
Died	35.7 (519)	35.8 (662)	35.7 (1181)
**Duration of critical care. Days, median (IQR)**			
Discharged patients	6 (4 - 10)	5 (3 - 9)	6 (3 - 9)
Deaths	13 (7 - 19)	11 (6 - 16)	11 (6 - 17)
**Organ support***			
No respiratory support	2.5 (36)	3.2 (59)	2.9 (95)
Advanced respiratory support	47.6 (692)	47.2 (873)	47.3 (1565)
Basic respiratory support	86.1 (1253)	82.0 (1518)	83.8 (2771)
No cardiovascular support	5.7 (83)	6.8 (126)	6.3 (209)
Advanced cardiovascular support	19.5 (284)	19.2 (355)	19.3 (639)
Basic cardiovascular support	93.0 (1353)	91.3 (1690)	92.0 (3043)
Renal support	13.6 (198)	12.0 (223)	12.7 (421)
Liver support	2.2 (32)	4.2 (78)	3.3 (110)
Neurological support	7.0 (102)	7.3 (135)	7.2 (237)
**Duration of organ support* Days, median (IQR)**			
Advanced respiratory support	11 (6 - 19)	9 (5 - 15)	10 (6 - 16)
Total (advanced + basic) respiratory support	8 (4 - 15)	7 (4 - 13)	8 (4 - 14)
Advanced cardiovascular support	2 (1 - 5)	2 (1 - 4)	2 (1 - 5)
Total (advanced + basic) cardiovascular support	8 (5 - 15)	7 (4 - 13)	8 (4 - 14)
Renal support	5.5 (3 - 10)	4.5 (2 - 9)	5 (2 - 9)

* Among patients who have been discharged or died.

Appendix, part 5: Demographic, medical characteristics and indicators of acute severity observed for patients in the critical care cohort who have completed their critical care outcome (discharged alive or dead).

	**Non-SGTF** *Cols% (counts)*	**SGTF** *Cols% (counts)*	**Full Cohort** *Cols% (counts)*
**Total number of patients**	1455	1851	3306
**Mean age (SD)**	59.2 (12.8)	57.7 (12.7)	58.4 (12.7)
**Sex**			
Female	32.6 (475)	33.4 (619)	33.1 (1094)
Male	67.4 (980)	66.6 (1232)	66.9 (2212)
**Ethnicity**			
White	72.8 (1059)	68.2 (1262)	70.2 (2321)
Indian	4.0 (58)	3.8 (71)	3.9 (129)
Pakistani	6.3 (91)	4.8 (89)	5.4 (180)
Bangladeshi	1.5 (22)	2.0 (37)	1.8 (59)
Other Asian	4.1 (59)	4.2 (78)	4.1 (137)
Caribbean	0.8 (12)	1.4 (26)	1.1 (38)
Black African	0.8 (11)	1.7 (31)	1.3 (42)
Chinese	0.5 (7)	0.4 (8)	0.5 (15)
Other ethnic group	4.2 (61)	5.2 (97)	4.8 (158)
Not recorded	5.2 (75)	8.2 (152)	6.9 (227)
**Prior length of hospital stay [N =1834/1454]**			
Mean (SD)	2.5 (13.4)	2.9 (17.0)	2.7 (15.5)
Median IQR	1 (0 - 3)	1 (0 - 3)	1 (0 - 3)
**Dependency before admission to acute hospital care**			
Able to live without assistance in daily activities	88.4 (1286)	86.6 (1603)	87.4 (2889)
Some assistance in daily activities	8.7 (126)	7.1 (131)	7.8 (257)
Total assistance with all daily activities	<5	<5	0.2 (6)
Not recorded	2.8 (41)	6.1 (113)	4.7 (154)
**Severe comorbidities**			
Cardiovascular	<5	>10	0.4 (13)
Respiratory	0.5 (8)	0.9 (16)	0.7 (24)
Renal*	0.5 (8)	0.3 (6)	0.4 (14)
Liver	>5	<5	0.3 (9)
Metastatic disease	>5	<5	0.3 (9)
Haematological malignancy	0.8 (12)	0.4 (7)	0.6 (19)
Immunocompromised	2.1 (31)	1.3 (24)	1.7 (55)
**BMI**			
<25	22.2 (323)	26.3 (487)	24.5 (810)
25 to <30	28.1 (409)	26.1 (484)	27.0 (893)
30 to <40	37.7 (549)	34.4 (637)	35.9 (1186)
40+	12.0 (174)	13.1 (243)	12.6 (417)
**CPR within previous 21h**			
In the community	0.8 (11)	0.4 (7)	0.5 (18)
In the hospital	0.7 (10)	0.9 (16)	0.8 (26)
No	97.5 (1418)	96.1 (1778)	96.7 (3196)
Not recorded	1.1 (16)	2.7 (50)	2.0 (66)
**Currently or recently pregnant**			
Currently pregnant	0.5 (7)	0.8 (15)	0.7 (22)
Recently pregnant (within 6 weeks)	0.3 (5)	0.5 (10)	0.5 (15)
Not known to be pregnant	99.2 (1443)	98.6 (1826)	98.9 (3269)
**Invasively ventilated within first 24h**			
No	74.8 (1088)	64.8 (1200)	69.2 (2288)
Yes	22.5 (327)	25.2 (467)	24.0 (794)
Not recorded	2.7 (40)	9.9 (184)	6.8 (224)
**APACHE II score [N = 1540/2869]**			
Mean (SD)	13.9 (5.1)	12.9 (4.9)	13.3 (5.0)
Median (IQR)	13 (11 - 16)	13 (10 - 16)	13 (10 - 16)

**PaO2/FiO2 ratio [N = 1329/1540]**			
Median (IQR)	13.3 (10.0 – 18.2)	13.5 (9.9 – 18.3)	13.4 (9.8 – 18.2)
**PaO2/FiO2 ratio**			
< 13.3 kPa (<100 mmHg)	45.9 (668)	40.2 (745)	42.7 (1413)
13.3-16.6 kPa (100-200 mmHg)	38.1 (554)	36.3 (671)	37.1 (1225)
> 26.6 kPa (> 200 mmHg)	7.4 (107)	6.7 (124)	7.0 (231)
Not recorded	8.7 (126)	16.8 (311)	13.2 (437)
**FiO2 [N = 1329/1540]**			
Median (IQR)	0.6 (0.45 – 0.75)	0.6 (0.45 – 0.80)	0.6 (0.45 – 0.77)

* Chronic, irreversible renal disease and being dialysis dependent prior to critical care unit admission.

Appendix, part 6: Demographic, medical characteristics and indicators of acute severity observed in patients of the critical care-cohort after matching.

	**Non-SGTF** *Cols% (counts)*	**SGTF** *Cols% (counts)*	**Full Cohort** *Cols% (counts)*
**Total number of patients**	1031	1031	2062
**Mean age (SD)**	59.6 (12.6)	57.9 (12.4)	58.7 (12.5)
**Sex**			
Female	34.4 (355)	36.6 (377)	35.5 (732)
Male	65.6 (676)	63.4 (654)	64.5 (1330)
**Ethnicity**			
White	69.6 (718)	70.4 (726)	70.0 (1444)
Indian	4.1 (42)	3.0 (31)	3.5 (73)
Pakistani	4.2 (43)	6.5 (67)	5.3 (110)
Bangladeshi	1.4 (14)	1.8 (19)	1.6 (33)
Other Asian	2.9 (30)	3.0 (31)	3.0 (61)
Caribbean	0.9 (9)	0.7 (7)	0.8 (16)
Black African	0.8 (8)	1.6 (17)	1.2 (25)
Chinese	0.5 (5)	0.1 (1)	0.3 (6)
Other ethnic group	4.5 (46)	4.5 (46)	4.5 (92)
Not recorded	11.3 (116)	8.3 (86)	9.8 (202)
**Prior length of hospital stay [N =1008 / 1024]**			
Mean (SD)	3.4 (16.7)	2.4 (11.8)	2.9 (14.5)
Median IQR	1 (0 - 3)	1 (0 - 3)	1 (0 - 3)
**Dependency before admission to acute hospital care**			
Able to live without assistance in daily activities	77.1 (795)	81.3 (838)	79.2 (1633)
Some assistance in daily activities	9.1 (94)	6.8 (70)	8.0 (164)
Total assistance with all daily activities	<5	<5	0.3 (6)
Not recorded	13.5 (139)	11.6 (120)	12.6 (259)
**Severe comorbidities [N = 3641/ 1761]**			
Cardiovascular	<5	>5	0.5 (10)
Respiratory	1.4 (14)	1.0 (10)	1.2 (24)
Renal*	<5	0.7 (7)	0.4 (9)
Liver	<5	<5	<5
Metastatic disease	>5	<5	0.4 (8)
Haematological malignancy	>5	<5	0.5 (11)
Immunocompromised	2.1 (22)	1.6 (16)	1.8 (38)
**BMI**			
<25	33.8 (348)	30.3 (312)	32.0 (660)
25 to <30	19.8 (204)	24.4 (252)	22.1 (456)
30 to <40	35.0 (361)	33.4 (344)	34.2 (705)
40+	11.4 (118)	11.9 (123)	11.7 (241)
**CPR within previous 21h**			
In the community	0.6 (6)	0.3 (3)	0.4 (9)
In the hospital	1.2 (12)	0.7 (7)	0.9 (19)
No	90.2 (930)	92.0 (949)	91.1 (1879)
Not recorded	8.1 (83)	7.0 (72)	7.5 (155)
**Currently or recently pregnant**			
Currently pregnant	<5	0.5 (5)	0.4 (8)
Recently pregnant (within 6 weeks)	0.5 (5)	0.9 (9)	0.7 (14)
Not known to be pregnant	99.2 (1023)	98.6 (1017)	98.9 (2040)
**Invasively ventilated within first 24h**			
No	57.6 (594)	61.3 (632)	59.5 (1226)
Yes	28.1 (290)	23.9 (246)	26.0 (536)
Not recorded	14.3 (147)	14.8 (153)	14.5 (300)
**APACHE II score [N = 922/913]**			
Mean (SD)	13.7 (5.6)	13.0 (4.9)	13.4 (5.3)

Median (IQR)	14 (11 - 16)	13 (10 - 16)	13 (10 - 16)
**PaO2/FiO2 ratio [N = 820/812]**			
Median (IQR)	13.2 (9.6 – 18.2)	13.3 (9.8 – 17.7)	13.2 (9.8 – 18.0)
**PaO2/FiO2 ratio**			
< 13.3 kPa (<100 mmHg)	40.8 (421)	39.5 (407)	40.2 (828)
13.3-16.6 kPa (100-200 mmHg)	31.1 (321)	35.3 (364)	33.2 (685)
> 26.6 kPa (> 200 mmHg)	7.6 (78)	4.0 (41)	5.8 (119)
Not recorded	20.5 (211)	21.2 (219)	20.9 (430)
**FiO2 [N = 820/812]**			
Median (IQR)	0.6 (0.50 – 0.75)	0.6 (0.50 – 0.80)	0.6 (0.50 – 0.75)

* Chronic, irreversible renal disease and being dialysis dependent prior to critical care unit admission.

Appendix, part 7: Demographics characteristics of primary care patients tested positive in hospital and community settings between 1st November 2020 and 26th January 2021 by months.

	November 2020		*December 2020*		*January 2021*	
	**Hospital settings** *Cols% (counts)*	**Community settings** *Cols% (counts)*	**Hospital settings** *Cols% (counts)*	**Community settings** *Cols% (counts)*	**Hospital settings** *Cols% (counts)*	**Community settings** *Cols% (counts)*
**Total number of patients**	10520	76188	16999	134493	20520	171206
**Mean age (SD)**	57.3 (23.7)	38.2 (19.1)	55.9 (23.7)	37.3 (18.5)	57.0 (23.2)	39.3 (18.9)
**Deaths**	15.6 (1644)	0.7 (517)	15.8 (2678)	0.8 (1118)	16.0 (3284)	1.1 (1858)
**CCU admitted**	3.4 (355)	0.3 (241)	3.9 (657)	0.4 (484)	3.0 (617)	0.3 (647)
**SGTF**	0.0 (0)	6.8 (5199)	0.0 (0)	32.9 (44280)	0.0 (0)	40.0 (68448)
**Sex**						
Female	55.1 (5797)	54.2 (41301)	55.9 (9497)	54.4 (73123)	54.2 (11114)	54.2 (92871)
Male	44.9 (4723)	45.8 (34887)	44.1 (7502)	45.6 (61370)	45.8 (9406)	45.8 (78335)
**Age groups**						
18-29	15.9 (1677)	37.0 (28213)	16.4 (2789)	36.9 (49682)	14.8 (3033)	33.9 (58096)
30-39	11.3 (1188)	18.3 (13922)	12.6 (2143)	19.8 (26683)	12.5 (2568)	19.8 (33842)
40-49	11.4 (1195)	15.9 (12125)	12.4 (2114)	17.5 (23496)	11.8 (2416)	16.3 (27942)
50-59	13.1 (1378)	15.1 (11507)	13.4 (2275)	14.3 (19245)	13.5 (2767)	15.8 (26991)
60-69	10.8 (1136)	7.8 (5934)	10.4 (1767)	6.6 (8936)	11.7 (2398)	8.0 (13669)
70-79	13.7 (1443)	3.3 (2481)	12.4 (2107)	2.7 (3581)	13.5 (2775)	3.3 (5565)
80-99	23.8 (2503)	2.6 (2006)	22.4 (3804)	2.1 (2870)	22.2 (4563)	3.0 (5101)
**Ethnicity**						
White	65.1 (6851)	61.7 (46995)	60.8 (10343)	58.8 (79116)	60.9 (12491)	57.3 (98076)
Indian	3.1 (330)	3.4 (2599)	3.7 (631)	4.1 (5523)	3.1 (635)	3.9 (6707)
Pakistani	2.9 (303)	4.4 (3358)	2.5 (419)	3.4 (4640)	2.3 (467)	3.3 (5702)
Bangladeshi	1.5 (161)	2.8 (2105)	2.4 (415)	3.9 (5194)	1.9 (389)	3.4 (5778)
Other Asian	1.8 (193)	1.9 (1428)	2.8 (481)	2.5 (3315)	2.6 (530)	2.6 (4392)
Caribbean	1.1 (120)	0.5 (413)	2.1 (350)	1.1 (1460)	2.7 (547)	1.5 (2578)
Black African	2.3 (238)	1.8 (1359)	3.7 (634)	2.4 (3206)	4.0 (826)	3.0 (5127)
Chinese	0.3 (36)	0.2 (134)	0.3 (52)	0.4 (501)	0.3 (58)	0.3 (580)
Other ethnic group	3.1 (325)	3.3 (2517)	4.6 (779)	4.8 (6425)	5.0 (1016)	4.8 (8282)
Not recorded	18.7 (1963)	20.1 (15280)	17.0 (2895)	18.7 (25113)	17.4 (3561)	19.8 (33984)
**House size**						
1 person	38.9 (4091)	26.2 (19924)	39.0 (6634)	25.3 (33981)	41.2 (8460)	27.3 (46674)
2 people	23.9 (2518)	20.0 (15271)	23.8 (4045)	19.0 (25614)	23.6 (4844)	20.5 (35027)
3-5 people	29.0 (3046)	45.0 (34281)	29.6 (5037)	47.2 (63519)	28.1 (5765)	43.6 (74676)
6-10 people or more	8.2 (865)	8.8 (6712)	7.5 (1283)	8.5 (11379)	7.1 (1451)	8.7 (14829)
**House type**						
Neither	95.6 (10054)	98.9 (75313)	96.4 (16393)	99.0 (133214)	96.5 (19795)	98.7 (168939)
Carehome	4.1 (434)	1.1 (818)	3.2 (547)	0.9 (1168)	3.2 (662)	1.2 (2076)
Homeless	0.3 (32)	0.1 (57)	0.3 (59)	0.1 (111)	0.3 (63)	0.1 (191)
**BMI**						
<25	60.2 (6334)	54.6 (41572)	60.9 (10348)	55.7 (74926)	58.9 (12086)	55.6 (95255)
25-30	15.8 (1663)	12.8 (9779)	15.2 (2582)	12.0 (16074)	16.5 (3376)	12.9 (22168)
30-40	7.2 (761)	5.3 (4021)	7.1 (1212)	4.8 (6395)	7.5 (1545)	5.4 (9293)
>= 40	3.9 (413)	3.0 (2284)	3.8 (650)	2.5 (3362)	4.3 (878)	2.9 (4940)
Not recorded	12.8 (1349)	24.3 (18532)	13.0 (2207)	25.1 (33736)	12.8 (2635)	23.1 (39550)

**Smoking status**						
Non smoker	56.0 (5889)	56.6 (43127)	57.5 (9777)	56.0 (75380)	56.5 (11589)	55.5 (95012)
Ex smoker	27.8 (2924)	17.5 (13357)	25.6 (4357)	17.1 (22994)	25.9 (5312)	17.5 (30027)
Light smoker	7.9 (826)	8.3 (6319)	8.1 (1385)	8.5 (11474)	9.5 (1956)	10.8 (18491)
Moderate smoker	1.7 (175)	1.5 (1162)	1.6 (275)	1.4 (1914)	1.9 (396)	2.1 (3554)
Heavy smoker	1.0 (109)	0.5 (388)	0.9 (156)	0.5 (617)	1.0 (210)	0.8 (1311)
Not recorded	5.7 (597)	15.5 (11835)	6.2 (1049)	16.4 (22114)	5.2 (1057)	13.3 (22811)
**Geographical region**						
East Midlands	1.8 (186)	2.5 (1912)	1.5 (256)	1.5 (2045)	1.5 (299)	1.7 (2890)
East of England	2.5 (262)	2.0 (1549)	4.2 (721)	3.9 (5229)	4.1 (844)	3.7 (6385)
London	16.1 (1694)	19.5 (14841)	32.1 (5460)	40.5 (54499)	30.6 (6287)	33.4 (57120)
North East	4.2 (447)	3.8 (2886)	2.1 (364)	1.4 (1895)	2.0 (411)	1.6 (2654)
North West	26.6 (2798)	23.1 (17575)	14.6 (2480)	10.6 (14302)	17.4 (3575)	17.5 (29880)
South Central	7.8 (822)	8.4 (6423)	11.2 (1910)	11.2 (15070)	13.2 (2701)	11.7 (20001)
South East	10.6 (1115)	10.8 (8264)	15.2 (2580)	16.9 (22754)	10.9 (2237)	11.8 (20281)
South West	11.3 (1185)	9.9 (7545)	7.2 (1232)	4.9 (6590)	7.8 (1598)	6.3 (10775)
West Midlands	14.8 (1553)	14.8 (11304)	9.3 (1575)	7.3 (9845)	10.3 (2120)	10.6 (18124)
Yorkshire & Humber	4.4 (458)	5.1 (3889)	2.5 (421)	1.7 (2264)	2.2 (448)	1.8 (3096)
**Deprivation quintile**						
1 (least deprived)	21.0 (2214)	21.6 (16442)	18.7 (3187)	18.7 (25147)	17.7 (3641)	17.4 (29743)
2	20.9 (2202)	22.2 (16921)	20.0 (3399)	21.1 (28324)	19.6 (4027)	20.2 (34500)
3	22.1 (2325)	21.5 (16404)	21.4 (3632)	20.3 (27256)	21.0 (4301)	21.3 (36383)
4	20.7 (2174)	19.2 (14656)	20.5 (3488)	19.6 (26400)	20.8 (4274)	20.9 (35862)
5 (most deprived)	14.8 (1559)	14.8 (11295)	18.8 (3198)	19.7 (26466)	20.3 (4164)	19.6 (33625)
Not recorded	0.4 (46)	0.6 (470)	0.6 (95)	0.7 (900)	0.6 (113)	0.6 (1093)
**Comorbidities**						
Asthma	15.8 (1667)	15.2 (11561)	14.6 (2482)	14.4 (19379)	15.4 (3155)	14.4 (24617)
COPD	8.0 (838)	1.2 (918)	6.8 (1159)	0.8 (1120)	6.5 (1343)	1.2 (1994)
Diabetes type 1	1.1 (120)	0.5 (403)	0.9 (145)	0.6 (772)	0.8 (158)	0.5 (911)
Diabetes type 2	16.4 (1729)	5.0 (3843)	17.2 (2931)	4.5 (6051)	17.5 (3586)	5.5 (9378)
Hypertension	33.6 (3533)	10.9 (8333)	32.9 (5588)	9.6 (12974)	34.2 (7020)	11.5 (19739)
Parkinson	1.4 (146)	0.1 (108)	1.0 (177)	0.1 (181)	1.2 (245)	0.2 (323)
Epilepsy	2.5 (265)	1.2 (935)	2.1 (358)	1.1 (1498)	2.3 (482)	1.3 (2205)
Cerebral palsy	0.3 (27)	0.1 (85)	0.1 (25)	0.1 (126)	0.3 (57)	0.1 (226)
MND	0.1 (6)	0.0 (3)	0.0 (6)	0.0 (8)	0.0 (5)	0.0 (6)
Huntington	0.0 (2)	0.0 (6)	0.0 (2)	0.0 (12)	0.0 (6)	0.0 (22)
Multiple sclerosis	0.4 (43)	0.2 (122)	0.4 (67)	0.1 (165)	0.3 (68)	0.2 (283)
Myasthenia	0.1 (11)	0.0 (21)	0.1 (18)	0.0 (31)	0.1 (12)	0.0 (43)
Downs syndrome	0.1 (13)	0.0 (31)	0.1 (18)	0.1 (68)	0.2 (38)	0.1 (130)

Learning disabilities	3.8 (395)	1.8 (1404)	3.0 (510)	1.6 (2210)	3.8 (771)	2.0 (3355)

Appendix, part 8: Counts of deaths by region compared with the ones observed in the primary care cohort. The counts of deaths in England were taken from https://coronavirus.data.gov.uk/details/deaths. All regions were covered in the primary care cohort for both the SGTF and non-SGTF group.

	**England (gov.uk data)**	**Primary care cohort**	**Coverage in the study**	**Primary care cohort**	
	Cols% (counts)	*Cols% (counts)*	*Row %*	Non- SGTF	SGTF
Number of deaths	65494	899	1.4	334	
**Geographical region**					
East Midlands	9.4 (6174)	1.5 (13)	0.2	2.1 (7)	1.1 (6)
East of England	13.7 (8561)	2.0 (18)	0.2	<5	>10
London	13.2 (8650)	17.5 (157)	1.8	6.9 (23)	23.7 (134)
North East	4.7 (3088)	6.2 (56)	1.8	12.0 (40)	2.8 (16)
North West	14.0 (9203)	30.1 (271)	2.9	39.9 (133)	24.4 (138)
South Central		9.6 (86)		4.5 (15)	12.6 (71)
South East	17.5 (11503)	11.2 (101)	0.9	3.3 (11)	15.9 (90)
South West	6.9 (4507)	3.7 (33)	0.7	5.4 (18)	2.7 (15)
West Midlands	12.0 (7854)	14.7 (132)	1.7	18.3 (61)	12.6 (71)
Yorkshire & Humber	9.1 (5954)	3.6 (32)	0.5	6.6 (22)	1.8 (10)

Appendix, part 8: Counts of deaths observed in the primary care cohort by age group and COVID19 28-day mortality HR by age groups. Late update for mortality data was 23rd February 2021.

	**Non-SGTF** *Within age group deaths % (counts)*	**SGTF** *Within age group deaths % (counts)*	**HR (95% CI)** *Baseline (50-59)*
**Age group**			
18-29	<5	<5	0.005 (0.0007, 0.03)
30-39	0.0 (7)	0.0 (12)	0.15 (0.09, 0.25)
40-49	0.0 (7)	0.1 (24)	0.26 (0.18, 0.39)
50-59	0.3 (34)	0.4 (76)	2.80 (2.19, 3.58)
60-69	0.8 (48)	1.3 (113)	1
70-79	3.3 (83)	4.8 (145)	10.82 (8.50, 13.76)
80-99	12.2 (155)	15.0 (194)	37.72 (29.31, 48.54)
